# Primary Intramedullary Histiocytic Sarcoma with Leptomeningeal Dissemination: Imaging, Pathologic, and Molecular Correlation

**DOI:** 10.3390/diagnostics16162623

**Published:** 2026-08-18

**Authors:** Su Hong Kim, Hee Jung Kwon, Mi Jin Gu, Wook Tae Park

**Affiliations:** 1 Department of Radiology, Yeungnam University College of Medicine, Daegu 42415, Republic of Korea; drshkim@yu.ac.kr; 2 Department of Pathology, Yeungnam University College of Medicine, Daegu 42415, Republic of Korea; hjs336@ynu.ac.kr (H.J.K.); mjgu@yu.ac.kr (M.J.G.); 3 Department of Orthopedics, Yeungnam University College of Medicine, Daegu 42415, Republic of Korea

**Keywords:** histiocytic sarcoma, intramedullary spinal cord tumor, leptomeningeal dissemination, TP53

## Abstract

Central nervous system histiocytic sarcoma is exceedingly uncommon, particularly with intramedullary spinal cord involvement. We describe a 27-year-old man presenting with motor weakness and radiating pain. MRI revealed a homogeneously enhancing intramedullary mass at the C2–3 level with diffuse leptomeningeal dissemination, raising suspicion for a disseminated neoplastic process. Histopathology demonstrated pleomorphic large histiocyte-like cells with strong CD163, CD68, and lysozyme expression, confirming histiocytic sarcoma. Targeted next-generation sequencing detected a TP53 hotspot mutation (p.Arg273His), classified as a Tier II variant according to the joint AMP/ASCO/CAP guidelines, while additional variants were of uncertain clinical significance. This case highlights the rare intramedullary presentation of histiocytic sarcoma with leptomeningeal dissemination and emphasizes the importance of radiologic–pathologic correlation for accurate diagnosis.

**Figure 1:**
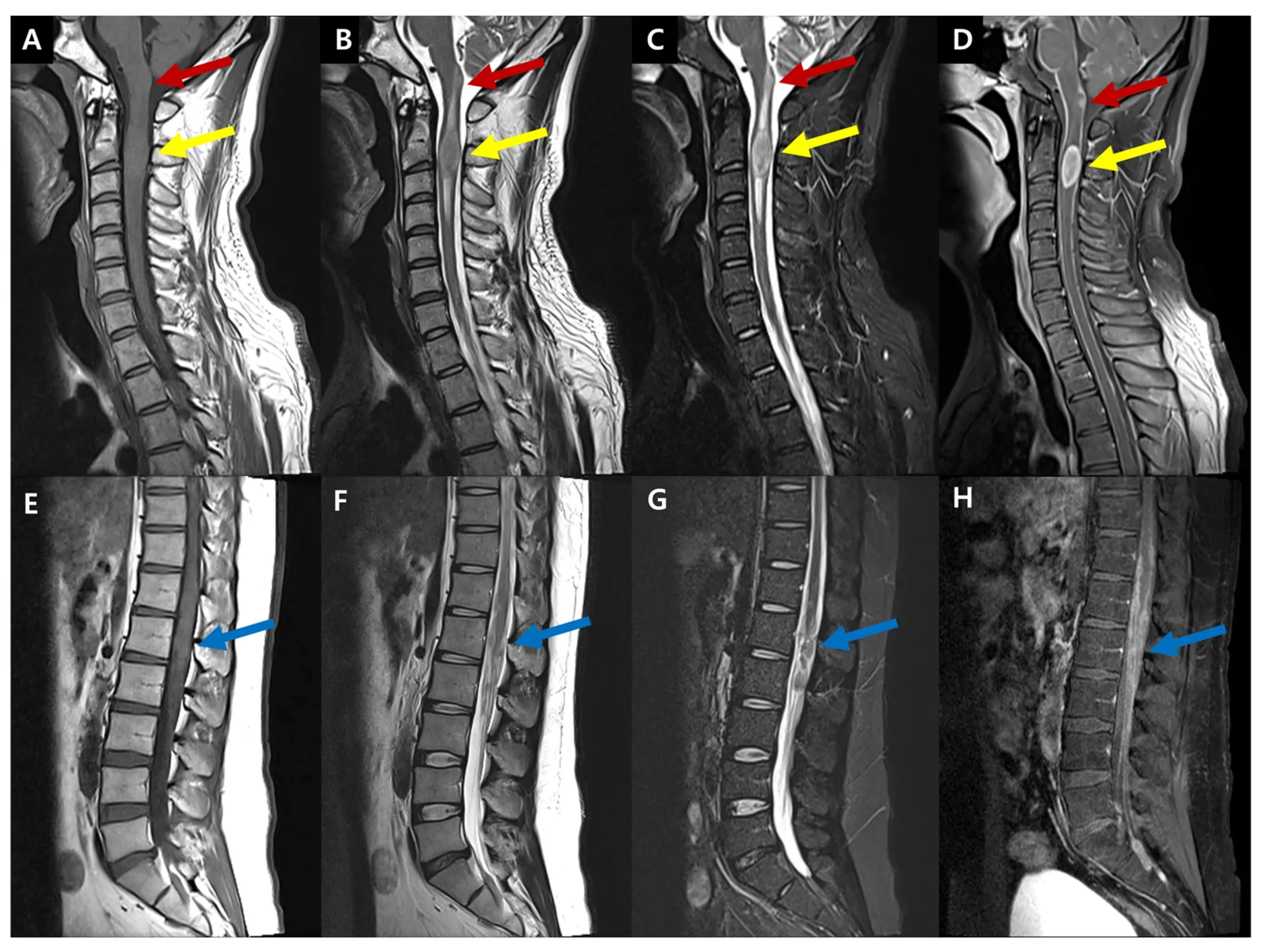
A 27-year-old man presented with motor weakness and radiating pain throughout the body. He had no history of hematologic malignancy, and routine laboratory evaluation was unremarkable. Brain, cervical spine, and lumbar spine MRI examinations were performed. Sagittal T1-weighted (**A**), T2-weighted (**B**), and STIR (**C**) images of the cervical spine revealed an expansile intramedullary lesion at the C2–3 level (yellow arrows) associated with spinal cord swelling and syringomyelia. The lesion was relatively well-circumscribed, appearing isointense on T1-weighted imaging (**A**), mildly hyperintense on T2-weighted imaging (**B**), and hyperintense on STIR imaging (**C**). Contrast-enhanced sagittal T1-weighted imaging (**D**) demonstrated homogeneous enhancement of the intramedullary lesion (yellow arrows). An additional enhancing lesion at the C1 level (red arrows) suggested disseminated disease. Sagittal T1-weighted (**E**), T2-weighted (**F**), and STIR (**G**) images of the lumbar spine demonstrated diffuse leptomeningeal dissemination along the lower spinal cord and cauda equina (blue arrows). Sagittal contrast-enhanced T1-weighted imaging (**H**) exhibited linear and nodular enhancement along the lower spinal cord and cauda equina (blue arrows). Brain MRI additionally demonstrated diffuse leptomeningeal enhancement in the posterior cranial fossa. Initial staging was performed using contrast-enhanced whole-body CT, brain MRI, and whole-spine MRI. Contrast-enhanced whole-body CT demonstrated no extracranial involvement, while MRI confirmed disease confined to the central nervous system, supporting the diagnosis of primary central nervous system histiocytic sarcoma. Routine laboratory investigations, including complete blood counts and peripheral blood smear examination, were unremarkable. Bone marrow biopsy was not performed due to the absence of clinical or laboratory findings suggestive of systemic hematologic involvement.

**Figure 2:**
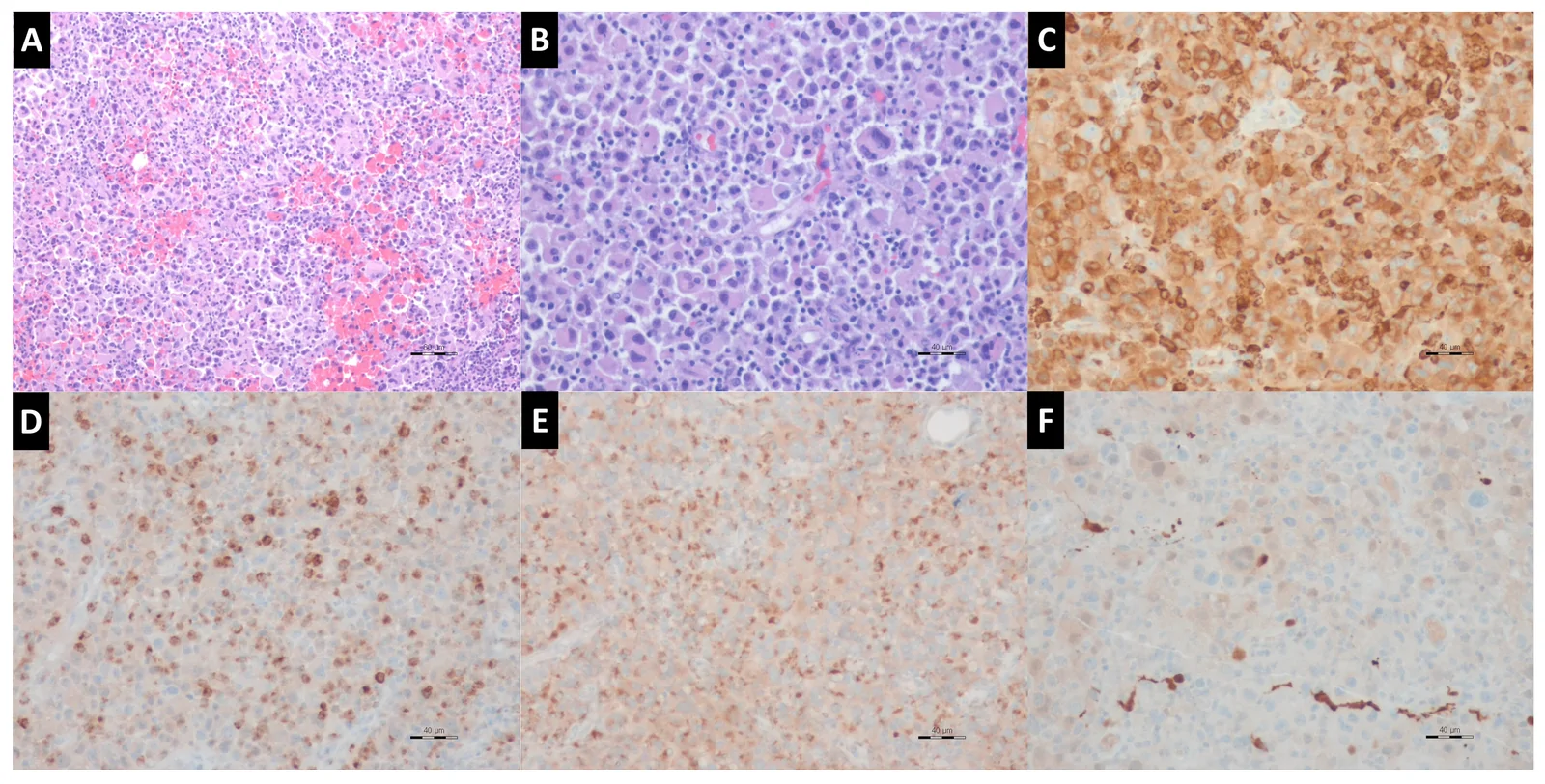
The patient underwent emergency surgical resection of the tumor due to progressive paralysis. The tumor demonstrated diffuse infiltration of the spinal cord parenchyma (**A**, ×100). The neoplastic cells were large and pleomorphic, with oval to eccentrically positioned nuclei, prominent nucleoli, and occasional nuclear grooves, resembling mature histiocytes (**B**, ×200). Immunohistochemistry revealed diffuse positivity for CD163 (**C**, ×100), lysozyme (**D**, ×100), and CD68 (**E**, ×200), with focal patchy S-100 protein expression in a subset of tumor cells (**F**, ×200). Additional immunohistochemical findings included LCA positivity, diffuse nuclear p53 overexpression, diffuse membranous CD4 positivity, and a high Ki-67 labeling index (~40%), whereas cytokeratin (AE1/AE3), ALK, myeloperoxidase, EMA, CD1a, CD21, CD30, CD43, CD117, and CD138 were negative. The diffuse nuclear overexpression of p53 was concordant with the TP53 hotspot mutation (p.Arg273His) detected by targeted next-generation sequencing. The final diagnosis was histiocytic sarcoma with leptomeningeal dissemination. Targeted next-generation sequencing using the TruSight Oncology 500 assay (1.94 Mb) identified the TP53 missense variant (c.818G>A, p.Arg273His) classified as Tier II according to the joint AMP/ASCO/CAP guidelines for cancer variant interpretation and reporting [1], with a variant allele frequency (VAF) of 30.3%. The tumor was microsatellite stable (MSS; MSI score, 6.5) and had a low tumor mutational burden of 2.3 mutations/Mb. Several Tier III variants of uncertain clinical significance were also detected, including FANCG (c.8C>T, p.Arg3Leu; VAF 66.8%), NOTCH4 (c.4063C>T, p.Arg1355Trp; VAF 55.5%), KIT (c.2866C>T, p.Arg956Trp; VAF 42.5%), BCR (c.1369G>A, p.Asp457Asn; VAF 42.4%), FLT1 (c.2878G>A, p.Val960Ile; VAF 36.8%), CCNE1 (c.694A>G, p.Met232Val; VAF 11.2%), and ARID1A (c.6116A>G, p.Gln2039Arg; VAF 7.7%). The TruSight Oncology 500 assay was performed using both DNA and RNA panels. RNA-based fusion analysis revealed no pathogenic kinase fusions involving ALK, ROS1, RET, or NTRK. Histiocytic sarcoma is a rare, aggressive hematolymphoid malignancy characterized by morphologic and immunophenotypic features of mature histiocytes. Primary central nervous system involvement is exceedingly uncommon, with intramedullary spinal cord disease being particularly rare [2,3]. Only a limited number of cases have been reported, and the radiologic findings are often non-specific, frequently mimicking primary spinal cord tumors, lymphoma, or inflammatory and infectious disorders [2,4]. In the present case, MRI revealed a homogeneously enhancing intramedullary lesion with diffuse leptomeningeal dissemination, suggesting a disseminated neoplastic or inflammatory process. Because leptomeningeal spread is more commonly associated with metastatic disease, lymphoma, or infectious meningitis, these imaging features can present a significant diagnostic challenge [3,5]. This case underscores the necessity of considering rare hematolymphoid malignancies, including histiocytic sarcoma, in the differential diagnosis of intramedullary spinal cord lesions with leptomeningeal involvement. The radiological differential included high-grade astrocytoma, ependymoma, primary central nervous system lymphoma, neurosarcoidosis, and infectious myelitis, which can all produce spinal cord enlargement and contrast enhancement. The presence of both homogeneous enhancement and diffuse leptomeningeal dissemination further limits radiologic specificity because these imaging features overlap with metastatic disease, lymphoma, and chronic infectious meningitis. Therefore, definitive diagnosis requires histopathologic examination supported by comprehensive immunophenotypic analysis [6]. The diagnosis of histiocytic sarcoma requires integrated histopathologic and immunophenotypic assessment. In our patient, the tumor demonstrated characteristic morphologic features, with large pleomorphic cells containing abundant eosinophilic cytoplasm and irregular nuclei, accompanied by strong expression of histiocytic markers, including CD163, CD68, and lysozyme. The lack of lineage-specific markers for epithelial, lymphoid, dendritic, and myeloid neoplasms helped exclude major diagnostic mimics. Focal S-100 positivity, as observed in this case, has been reported in histiocytic sarcoma and should not preclude the diagnosis in the appropriate immunophenotypic context [2,4]. The histopathologic differential diagnosis includes large B-cell lymphoma, anaplastic large cell lymphoma, myeloid sarcoma, Langerhans cell histiocytosis, Rosai–Dorfman disease, dendritic cell sarcomas, and poorly differentiated metastatic carcinoma [7]. Recent genomic studies indicate that histiocytic sarcoma is characterized predominantly by alterations in the RAS/RAF/MAPK signaling pathway, often accompanied by additional abnormalities involving CDKN2A and TP53. Recurrent kinase fusions involving ALK, ROS1, NTRK, and other genes have also been reported in a subset of histiocytic and dendritic cell neoplasms [4,8]. In the present case, the TruSight Oncology 500 assay incorporated DNA sequencing, RNA-based fusion analysis, and copy-number variant assessment. No pathogenic gene fusions, clinically significant copy-number alterations, or CDKN2A deletions were detected. Targeted DNA sequencing detected a TP53 hotspot mutation (p.Arg273His), representing the most biologically relevant finding. The additional variants detected in FANCG, NOTCH4, KIT, BCR, FLT1, CCNE1, and ARID1A are not recognized as recurrent or disease-defining alterations in histiocytic sarcoma and were therefore considered variants of uncertain clinical significance. The absence of canonical MAPK pathway alterations further underscores the molecular heterogeneity of histiocytic sarcoma [4,8]. Currently, the molecular findings in this case do not modify the diagnostic approach, prognostic assessment, or treatment strategy. Rather, the identification of the TP53 hotspot mutation primarily supports the malignant phenotype of the tumor, whereas the remaining Tier III variants should be regarded as descriptive genomic findings pending further validation of their biological significance. No approved targeted therapy can currently be recommended based solely on the genomic alterations identified in this case. Postoperatively, the patient developed persistent tetraplegia and received corticosteroid therapy followed by adjuvant spinal radiotherapy. At 3-month follow-up, brain and spinal MRI demonstrated stable leptomeningeal disease without significant interval progression. Whole-body ^18^F-FDG PET/CT performed 4 months after surgery revealed no evidence of extracranial involvement. At the last follow-up, the patient remained tetraplegic and was undergoing comprehensive rehabilitation. Overall, this case represents a rare intramedullary presentation of histiocytic sarcoma with diffuse leptomeningeal dissemination and emphasizes the diagnostic challenges associated with non-specific imaging findings. Accurate diagnosis requires an integrated assessment of radiologic, histopathologic, immunophenotypic, and molecular findings, particularly in rare intramedullary lesions with overlapping radiologic and pathologic differentials.

## Data Availability

The original contributions presented in this study are included in the article. Further inquiries can be directed to the corresponding author.

## Abbreviations

The following abbreviations are used in this manuscript:

MRI	Magnetic Resonance Imaging
STIR	Short Tau Inversion Recovery
CD	Cluster of Differentiation
LCA	Leukocyte Common Antigen
ALK	Anaplastic Lymphoma Kinase
EMA	Epithelial Membrane Antigen
Ki-67	Ki-67 Proliferation Index
TP53	Tumor Protein p53
FANCG	Fanconi Anemia Complementation Group G
NOTCH4	Neurogenic Locus Notch Homolog 4
KIT	KIT Proto-Oncogene, Receptor Tyrosine Kinase
BCR	Breakpoint Cluster Region
FLT1	Fms-Related Tyrosine Kinase 1 (VEGFR1)
CCNE1	Cyclin E1
ARID1A	AT-Rich Interaction Domain 1A
NGS	Next-Generation Sequencing
VAF	Variant Allele Frequency
RAS	Rat Sarcoma Viral Oncogene
RAF	Rapidly Accelerated Fibrosarcoma
MAPK	Mitogen-Activated Protein Kinase
CDKN2A	Cyclin-Dependent Kinase Inhibitor 2A
